# Chinese Self, Australian Other: Chinese as a Foreign Language Teacher Identity Construction in Australian Contexts

**DOI:** 10.3389/fpsyg.2021.792004

**Published:** 2021-12-15

**Authors:** Yu Han, Xiaoyan Ji

**Affiliations:** ^1^College English Education Center, Nanfang College, Guangzhou, China; ^2^School of Education, The University of Sydney, Sydney, NSW, Australia

**Keywords:** CFL teachers, teacher identity, Chinese self, Australian other, social whole

## Abstract

Research in the field of Chinese as a foreign language (CFL) education has been increasing in the past decades. However, the number of studies on CFL teacher identity is limited. To bridge the gap, this study employed a qualitative method to explore Chinese CFL teachers’ identity formation and reformation in Australian contexts. A Chinese-Australian language program was studied to examine the challenges, struggles and developments of Chinese CFL teachers who came to Australia to pursue professional growth. Five Master’s theses and three interview participants were included to paint a picture of how Chinese CFL teachers interact internally and externally with a new environment. Guided by Mead’s theory of self and other, we found that Chinese CFL teachers’ identity formation and reformation in Australian classrooms are deeply influenced by their self-identification and their integration with others in the community. Cultural connectedness is a key for organizational attitudes in the relationship of self and other. Chinese CFL teachers were found lacking the wholeness of self in Australian contexts, which led to obstacles in teacher identity construction. Insufficient communication between self and other resulted in their positioning crisis.

## Introduction

Internationalization in education has brought an increase in global teacher mobility (Sun, 2012). Chinese as a Foreign Language (CFL) education is one of the key sectors following the trend due to the growing demand for Chinese language education (Wang et al., 2013). Accordingly, there is a high demand for CFL teachers to satisfy the need. However, the fact is that “teacher shortage is one of the most significant challenges that policymakers have to face” (Ma et al., 2017, p. 824). Chinese native speakers are the main teacher force to satisfy the need. However, moving across cultures with divergent educational systems, CFL teachers face new problems and issues, which also offer chances for shaping them to become international language teachers. Professional identities and beliefs of foreign language teachers have been studied much in general, but not much attention has been paid to Chinese language teacher identity entering into Western educational contexts (Gao, 2010; Wang and Du, 2014). CFL teachers’ professional identity issue is one of the key fields under-researched.

Chinese as a foreign language teachers play a vital role in the implementation of CFL curricula and improvement of the effectiveness of Chinese language teaching. Therefore, professional development should be a priority in CFL research (Gao et al., 2014). However, the fact is that there are insufficient studies on teacher development in the area. Gong et al. (2020) reviewed CFL literature published by leading journals in mainland China from 2014 to 2018 and revealed that only five out of 60 empirical studies focused on teacher development, mainly in university contexts on in-service teachers rather than pre-service teachers. Comparatively, international studies on CFL teacher development has been increasing since 2004, when the first Confucius Institute was established in South Korea. However, international publications in the CFL teacher development field are few despite an increasing number of studies on CFL education, focusing more on content teaching and learning (Pinyin for instance) as well as students’ learning attitudes (Gong et al., 2018).

In Australia, CFL teachers have been in continuous demand, because the number of language learners has increased more than five times in the last 50 years and language accessibility is increasingly regarded as a factor for all-around development (Black et al., 2018). Communities are shifting from a traditional monolingual mindset to language-integrated education ideology (Fielding, 2016). Thus, many schools invest affluently in language promotion to satisfy the need and to foster globally competitive citizens. CFL learning has been soaring and ranks top five among foreign languages since 2015 (Orton, 2016). However, the need for CFL teachers cannot be met domestically. Fortunately, the burgeoning CFL market has driven vigorous development of teacher professionalization and mobility across cultures. CFL teachers from China meet part of the demand and become transnational language teachers, during which process their teacher identity has been shaped and reshaped mainly under the influence of divergent cultural factors. Against this background, it would be beneficial to investigate the roles that these transnational CFL teachers and others play in their construction of teacher identity in this unfamiliar cultural and educational context, which might offer a tiny hint to future CFL teacher education globally.

## Literature Review

### Theoretical Framework

Mead’s self and other theory is the theoretical foundation of this study. While examining self, Mead (1934) suggested that it is something of a developmental characteristic. Resultant of social experiences and relations, the individual objectifies self to complete a reflexive conversation. By being an object to itself, self enters impersonal reasoning of communication. It is the sort of communication with significant symbols that features the engagement of not only others but also individuals themselves. Conversing with self about what others are saying, one develops awareness of what they are going to say. Self and other communicate dynamically by community norms. Instead of operating self to demonstrate individual differences, recognizability of self is key for one to fit in and combine with other (Larrain and Haye, 2019). Ideological engagement occurs through the process of discourse where dialogical and dynamic meanings are communicated across boundaries. Mead and Deegan (2017) reported the role and value of self in social engagement and its positioning in education. Self-individuation takes on various forms with subjective agencies and objective consequences that lead to multiplicity of identity formation. Thibault (2019) coined the term “selving” to represent both entity and activity of self, indicating interdependent selves and multilateral influences.

In our mechanism, there is a tendency to view ourselves through others’ perspectives and to address ourselves like others do (Mead, 1934). As a consequence, others’ attitudes are taking over unconsciously and we use them in our actions. This is of great importance in the realization of self-consciousness. Different from animals that do not think from others’ perspectives, we take in others’ responses and make them our own experiences with specific meanings. Others’ responses can be called as a stimulus for our acts. Mead termed the mechanism “thought” and insisted that controlling one’s actions regarding others’ participation is key for thought. Social processes that involve communications are responsible for the construction and development of new thoughts. Meaning, instead of consciousness conceived outside experience, exists entirely within social relations.

To understand one’s position in a society, one needs to be viewed regarding both “I” and “me” (Mead, 1934). In preparation of “I” and “me,” gestures arouse in individuals the attitudes of others; “The taking of all of those organized sets of attitudes gives him his ‘me;’ that is the self he is aware of” (Mead, 1934, p. 175). “Me” takes others’ attitudes, knows the consequences of actions, and assumes responsibility for situations. “I,” on the other hand, is the response one performs concerning the attitudes of others. Therefore, “I” and “me” are not in the same position, but they are parts of a whole. “I” reflects uncertainty and freedom, while “me” represents the organization of attitudes. They collectively form a personality in the process of social experience. The two different elements of self, “I” and “me,” bring about novelty and conscious responsibility, respectively.

In the understanding of the development of self, several theories were proposed to approach different aspects of it. Positioning Theory (Davies and Harré, 1990) focused on intergroup interaction. Consistent with Montiel and De Guzman (2011), Davies and Harré recognized multifaceted selves and negotiation of positions. Dialogical Self Theory by Hermans (2001) emphasized psychological process while self assumes various positions. “I-positions” was studied to reveal linkages between self-internalization and societal progress. Three assumptions were made that contribute to the Position Exchange Theory (Gillespie and Martin, 2014): society has multiple positions (Durkheim, 2014), social positions contribute to different perspectives (Ross and Nisbett, 2011), and people move between positions (Dreier, 2009).

We take the attitudes of others in controlling our responses; thus, “The organized community or social group which gives to the individual his unity of self may be called ‘the generalized other”’ (Mead, 1934, p. 154). To develop a full self, one must bring other’s attitudes into oneself, bring social processes into one’s experience, take the attitudes of all members of a social group toward others in the same group, take their attitudes toward social activities, and generalize all the attitudes as a whole to which actions are called for. In the process of attaining a complete self, the social whole is a prerequisite through which activities are organized about all individual attitudes. Generalized other, encompassing social processes and individual thinking, influences the conduct of each member in a society. Social bearings and systematic patterns are reflected in the building of self through other.

Targeting the understanding of cultural dialogue and its responsiveness toward social life, Bandlamudi (2015) found that the penetration of culture from within is believed to influence the achievement and multiplicity of “outsideness.” Self-consciousness and self-organization (Friston, 2018) were also important in explaining the generation and recognition of changes in environments and the awareness of the ever-changing self. Body movement in society and accumulated experience were studied about integrated mind and mediation of movement (Wagoner et al., 2015) where body and mind took contextual shapes and interacted in different positions.

Moving to a foreign society, language teachers have to adapt to the new environment in which self and other collaboratively contribute to the formation of their new identity. By this theoretical guidance, we intend to explore the value of CFL teachers’ self-identification and factors that impact it. To understand who they are and how they work, the individual and social identities they are assigned have to be accounted for. Transformative as these identities are (Weedon, 1987; Sarup, 1996), negotiations of identity across contexts define the formation and development of language teachers’ core identity, the one at play for self-identification.

### Teacher Identity

Teacher identity has been widely regarded as an important factor that influences the way teachers view themselves and, to a large extent, how they interact with students. It is and could be shaped and reshaped under the impact of various factors and in different contexts (Beauchamp and Thomas, 2009). Global mindset and intercultural capability play an important role in shaping language teacher identity (Neilsen and Weinmann, 2020). Based on Australian pre-service teacher programs to Chile, Neilsen and Weinmann demonstrated the role that teacher identity plays in responding to the diversity of multicultural classrooms; specifically, self-examination in the context of social, economic, and political similarities and differences impacts how they teach, and mutual in-depth understanding was believed to be a key for connecting the unfamiliar with the familiar. Thus, self-critical examination was promoted to increase intercultural capabilities and decrease biases, since the distance between self and other was not only geographical separation but also identity difference.

Administrative management and professional learning could also shape and reshape teacher identity. Bradford and Braaten (2018) investigated teacher evaluation processes and their impact on teacher identity, and found that rigid evaluation activities with bureaucratic agendas deteriorated teacher identity rather than facilitated professional growth: teachers who were consistently challenged to meet systematic orientations would experience demoralization. Teachers’ vision of high-quality teaching was part of their understanding of teacher identity, which contributed to professional practice and learning. If measurement tools fail to authentically reflect the diversity of teacher identity, they would produce biased information; thus, the degree of inclusiveness in the evaluation system is a key factor that defines the recognition and construction of teacher identity.

Identity could be shaped by recipients and contextual factors. Teacher identity, thus, is closely related to students and environments. From a sociological point of view, Miller (2009) pointed out that teacher identity must be accounted for with consideration of such factors as the discourse in self-representation and sociocultural contexts, which is consistent with Gee and Gee (2007) opinion that “it’s not what you say or even how you say it, it is who you are and what you are doing while you say it” (p. 3). Students’ degree in accepting or denying a teacher’s message could shape a teacher’s identity; contextual factors like workplace conditions, social demographics, curricula, and resources are also believed to play a powerful part in constructing teacher identity. Negotiation with these factors, thus, has been regarded as a transformational interaction that enables the systematic development of teacher identity. The shifting of focus from internal psychological process to external social communication reflects the practicality and malleability of identity in incorporating with varied subjects.

As for language teacher identity, Duff and Uchida (1997) noted:

*Language teachers and students in any setting naturally represent a wide array of social and cultural roles and identities: as teachers or students*, *as gendered and cultured individuals*, *as expatriates or nationals*, *as native speakers or non-native speakers*, *as content-area or TESL/English language specialists*, *as individuals with political convictions*, *and as members of families*, *organizations*, *and society at large* (p. 451).

Positioning of self and other in a complex social and cultural context is crucial for language teachers, because it involves power relationships and mainstream discourses that determine the legitimacy of their languages and teaching. This is in agreement with Peirce’s (1995) notion that identity is a site of struggle, which changes continuously.

### Chinese as a Foreign Language Teacher Identity

Divergent, if not contradictory, findings have been revealed in the field of CFL teacher identity studies in cross-cultural contexts. Some find that CFL teachers are impacted strongly by Chinese educational culture situating in a cross-cultural context, and have reported that under the influence of Confucianism their professional identities tend to be authoritarian and teacher-centered (Pratt et al., 1999; Moloney and Xu, 2015; Han and Han, 2019). While others report that transferring to another culture has resulted in CFL teachers’ transformation of professional identity from “a sage on the stage” to “a guide by the side” considering to cater students’ needs. However, this transformation did not bring good effects and put CFL teachers in a dilemma and self-questioning (Han et al., 2019). Wang and Du (2014) studied immigrant Chinese teachers’ professional identity transformation in an intercultural context (Danish) and found that the professional identity of Chinese teachers is shaped both by their previous experiences of teacher-student relationship in the Chinese culture and their new experience as teachers in the Danish culture.

Pedagogical and linguistic authority is an important theme in the study on CFL teacher identity. The interactions between native resources, such as Chinese textbooks and interests of new learners, reflect the negotiation of education systems (Li, 2016). Old-fashioned teachers, referring to those with the identity of traditional methods and values, need innovation through which new learners of no cultural familiarity can establish connectedness. In the understanding of self in new contexts, CFL teachers adapt pedagogically to fulfill roles and goals. Intercultural similarities and differences have to be viewed with an open mindset so that CFL teacher identity and learner identity contribute to each other’s growth, as Ma (2014) pointed out that “The purpose of learning about and from each other is not to lose one’s own identity or just to become the other” (p. 173). CFL education in Australia has been growing and becoming more diversified (Moloney and Xu, 2015); thus, there is no longer a fixed role for CFL teachers to play or a defined parameter on how Chinese is to be taught.

Teacher identity was identified as one of the key factors impacting CFL pedagogy in a book concentrating on exploration of innovative CFL pedagogy edited by Moloney and Xu (2016). In her chapter, Wang (2016) contended that CFL teachers need to develop their identity beyond school to see the relationship between Chinese and Australian societies, which is the basis for cultivating their students to gain learner autonomy beyond the classroom and to be life-long learners. Following the same path, Tsung and Hooper (2016) found that Chinese native speakers’ social network in Australia plays a major role in the development of linguistic competence and confidence. It is, therefore, reasonable to believe that CFL teachers’ social identity in Australian contexts contributes to their professional identity. Diaz (2016) approached teacher identity from the perspective of teaching resources and stressed the importance of active implementation in the selection and adaption of resources according to their availability, suitability, and readiness. Teacher identity in this respect includes the competence from decision-making to bridging resource gaps, which can also be viewed as the representation of one culture to approach another. In Tasker’s (2016) chapter, CFL teachers’ technological identity was discussed. Their innovative pedagogies and technological beliefs enable collaborations and flexibilities of a wider range than traditional teaching. Effective engagement of technology in the Australian universities promoted attendance, teamwork, and independent learning of CFL. Zhen Li, through conducting a life-history narrative study of three CFL teachers who had over 20 years teaching experience, found that successful teacher identity construction in Western-based school contexts requires “an effective blend of Eastern and Western cultural values and pedagogical practices” (2016, p. 177).

As a country of diverse cultural backgrounds, Australia has witnessed the complexity of teacher identity interaction and transformation. CFL teachers, as one of the groups, are situated in the context of multilateral negotiations of power relations and language ideologies (Moloney and Xu, 2015). Understanding of them and their teaching requires systematic comprehension of their experience. Previous studies have revealed cultural influence on CFL teachers’ professional identity where experiences from both cultures play a role in the development of their understanding of the relationship. However, there was no specific mention of how diverse cultural elements interact with one another in the formation of new subjectivity and why individuals’ sense of unity influences their behavioral tendency. Former studies also covered CFL teachers’ negotiation with foreign education systems and their identity diversification. While CFL teachers have been researched widely about how they learn to teach in foreign environments, little has been done to research CFL teachers’ sense of belonging and its impact on their teaching. CFL teachers’ confidence has been studied in such areas as social network and learner autonomy; however, purposeful conversation between CFL teachers and their colleagues has not received study in depth. Therefore, we would like to argue that research in CFL teachers’ social organization of self is lacking in the field, leading to insufficient understanding of their struggles of positioning in cross-cultural contexts and identity construction and crisis. This study aims to add to the knowledge of how CFL student-teachers of Chinese background shape and reshape their professional identity in Australian contexts and its theoretical implications of self and other.

## Materials and Methods

### Program Setting

A Chinese-Australian bilateral provincial program is the focus of this study. Each year, a group of graduates (around 10) from Chinese universities (mostly in the same province due to the local government’s promotion of the program) is recruited to come to Sydney pursuing a Master’s degree in Education. Research-based as it is, the 2-year study includes practices of teaching Chinese in Western Sydney primary and secondary schools. As a start of the program, the graduates, who predominantly majored in Teaching Chinese as a Foreign Language and English as a Foreign Language (the English degree in Chinese universities refers to learning English language and culture as a major), need to take up International English Language Test System (IELTS), designed by the University of Cambridge and measures a person’s ability to listen, speak, read, and write English at the level that is necessary to go to universities mainly in Britain, Australia, Canada, and New Zealand where English is the native language, to ensure a reasonable English proficiency. There is a series of workshops to inform the candidates of Australian culture and potential environments. After arriving at the university in Sydney, the CFL teacher candidates undertake another series of workshops where teaching and culture are specified regarding local schools. Allocation to schools was random, depending partially on accommodation distance and transportation availability.

### Data Collection and Analysis

Eight participants of this study were from the above-mentioned program. Five of them are indirect participants, which means they did not participate in the study in person; instead, their reflective journals from their theses are resorted to as research data for this study. The other three participants, two males and one female, were involved in this study in person, from whom interview data were collected. One interviewee was enrolled in 2016 and the other two in 2017. They volunteered to participate in this study and liked to share their experiences. Formal consent forms were signed by the three interview participants.

Data for this study cover both theses data and interview data, which contribute to data triangulation (Maxwell, 2008). As for the theses data, the authors went through a total 17 theses of the program between 2013 and 2017 (the recent 5 years when data for this study were collected) and selected five of them with reflective journals that were most relevant to the focus of the study (one in 2013, one in 2015, and three in 2016). In terms of interview data, one author conducted all the semi-structured interviews in 2018, while the other author collated transcripts. Face-to-face interviews were employed in Chinese, as both the interviewer and the interviewees are native Chinese speakers. Terms, such as “different,” “respect,” “authority,” and “manage,” were frequently used in conversations. Each participant was interviewed twice with a 2-week gap, and each session lasted 50–70 min, totaling 372 mins.

The reason why there was a gap between interview and reflective journal data sources is that the CFL teachers who wrote the reflective journals in their theses had graduated and went back to China while the data for this study were being collected; the interview data came from another three participants from the same program who were conducting their CFL teaching and studying at the time and, thus, were available for interview participation. It is believed that this gap would not harm the reliability and validity of the study, because the same linguistic and cultural backgrounds they come from and the same linguistic and cultural contexts they enter into would bring them similar experiences and issues. The quotes demonstrated in the “Findings” section were both from reflective journals in their theses and interviews, with the interview data marked.

As a source of data, all the self-reflective journals in the CFL teachers’ thesis between 2013 and 2017 were resorted first as a general pool for consistency and randomness. It is noteworthy that only a few of them mentioned their struggles extensively as they were not the focus of their study. Accounts from candidates C1 to C5 were organized according to relevance toward the Chinese self, Australian student, and Australian environment (see Table 1 with key ideas from the theses). These are the variances manageable for the communication of two cultures and the basis of our organizational coding (Maxwell, 2008). Further elaboration of the organizational concepts (Richards and Morse, 2007) brought us to substantive implications of underlying themes that govern the direction of social interactions. After several rounds of discussion between the authors, it was believed that the prioritization of culture was key to self-identification and relationship with others; teaching skills played a conducive role as well. In the process of data organization, patterns emerged, and structures formed for themes and concepts to be further explained. The coding structure demonstrated not only categorical but also hierarchical connectedness. Self, other, and intercultural positionings were aligned dialogically with consideration of context and agency. How does Chinese thinking work in CFL teachers’ Australian teaching and how, in turn, understanding others in Australian contexts works for their Chinese thinking were the practical questions that we need to answer for our research questions. Here, Chinese thinking refers to CFL teachers’ internalized Chinese culture and its way to respond to other.

**TABLE 1:** Data coding (theses data).

Substantive implications	Organizational concepts (needs/strategies)	Key ideas
Learn Australian culture	Change Chinese self	• China and Australia were totally different (C2)
		• My pronunciation mistake caused a lot of misbehavior (C3)
		• Grammatical mistakes in the classroom instruction languages (C3)
		• I felt depressed and wanted to quit (C5)
		• I was afraid to give instructions (C5)
		• I was influenced by Chinese belief (C5)
		• I felt I was not a real teacher (C5)
		• My authority was ruined (C5)
		• I didn’t receive automatic respect (C1)
		• I doubted myself (C5)
		• I had never gained power in their eyes (C5)
		• I wanted to stand up and shout “get out of the door” (C1)
		• No hierarchy between teachers and students (C1)
		• Not sure if I should ignore it (misbehavior) (C1)
		• I was confused what instructive words to use (C1)
		• Cultural shock (C1)
Enforce Chinese culture	Change students	• They are not interested at all (C4)
		• They ignored my instruction (C5)
		• How could they be so rude (C5)
		• Totally out of control (C5)
		• They ignored me (C5)
		• No students greeting (C1)
		• No automatic respect (C1)
		• Whisper and distraction never ended (C5)
		• They were too noisy (C5)
		• Laughed with other students and talked without my permission (C5)
		• Students lost patience (C1)
Connect the two cultures	Change environments	• Without class teacher, I had to lift my voice (C5)
		• Classroom teacher took over the class (C4)
		• It’s like a vicious circle (C5)
		• Shocking classroom (C1)
		• Classroom environment not satisfying (C1)
		• Did not have positive classroom atmosphere (C1)

### Research Questions

To understand the dynamics related to the establishment of CFL teacher identity from the perspectives of self and other in Australian schools, this study targets the following research questions:

(1) What role does CFL teachers’ Chinese self play in the formation of their Australian teacher identity?
(2) How do social relations with Australian other affect CFL teacher identity?
(3) How to negotiate between self and other in Australian contexts for CFL teachers to develop a favorable teacher identity?

## Findings

Based on categorizations and connections of data, the findings of this study were structured into three sections to display the meaning of self-other relationship to CFL teachers in Australian classrooms, variant factors that contribute to different identities, and implications as well as results of different approaches.

### Self: Chinese Ideology That Impacts CFL Teacher Identity Construction

Chinese self refers to the self developed in China over time as the CFL teachers experienced the social network there. “Both subject and object” (Mead, 1934, p. 137), their Chinese self is a collection of social rules and interpersonal processes in which Chinese culture and customs are embedded. When exposed to Australian social structure and relationships, they experienced discomfort and disconnectedness to which most people referred as “culture shock.”

*I remember I had to work hard and listen to the teachers when I was a student*. *Therefore*, *it is to me that that should be the norm of the teacher-student relationship*. *It is in my culture*. *But I found students here wouldn’t care that much about you*. *In this culture*, *students do not listen to what you say*. *They do whatever they like and that causes conflicts*. *I feel I’m not a real teacher here* (*T1*, *interview data*).

“Culture” was T1’s keyword in describing troubles and his teacher identity was severely compromised. Consistent with the theses data key ideas (e.g., “I had no power” and “I felt I was not a teacher”), different attitudes from Australian students have caused issues on self-communication. The self they acquired from China in understanding education was that students need to show respect to teachers and that teachers possess unconditional authority. Although the CFL teachers were informed about the cultural differences beforehand, no one expected the reality to be “so bad” (T3) and it “hurts” (T1). All the participants mentioned the title “teacher” should at least have a certain meaning to students rather than being treated as “nothing” (T2). T2 viewed the cultural clash in a positive sense and took it as a chance to learn Australian culture. He thought it was fine to start from “zero” and believed in his “adaptability.” T1 said he did not feel good being a teacher in Australia and would look for other professions. He was weary of “surviving” when “so much have been paid” to be a teacher. T3 did not feel as bad because she had “low expectations” for both herself and the students from the beginning. There was no need for Australian teacher identity and her aim was to “survive.”

*I might not be respected; so what? I don’t really care about it that much*. *This is a journey to experience something and I want things to be natural*. *My goal is to finish the degree*. *When I go back to China*, *students there will respect me anyway* (*T3*, *interview data*).

### Other: Australian Factors That Contribute to Chinese as a Foreign Language Teacher Identity Formation

Students were regarded as the key “problem” (T1) that “burned” (T2) the CFL teachers out. As a major determinant of the CFL teacher’s Australian “me,” they displayed different ways through which teacher identity could be shaped. While “no respect” was the main concern of all the CFL teachers, they have different views toward it. These data mostly revealed frustration from gauging students with Chinese standards (e.g., “no greetings” and “ignored me” indicated in Table 1), while the interviews captured more subtleties. T3 believed students not only care less about her and Chinese but also “hate” school. She observed other classes and concluded that “that’s just how it is.” Some students “set up limits of what they are willing to do” (T2) and it challenged the CFL teachers’ ability to engage and interact. T1 was thrilled that his “silly joke worked, and the lesson went pretty good.” He prepared more interesting “stuff” and learned how to offer more variety. However, English was always a challenge for him both in giving instruction and random communication. T2 approached the students differently, although he also considered his English “not enough.” He worked on improving himself with challenges and wished to “make a change.” His goal was to go back to China and teach English in high school, so he valued this “opportunity” deeply and wanted to “learn something to bring back there.” T2 observed local teachers’ lessons and realized “their English was not complicated at all but very smooth, so it’s more of how you use it.” He admitted, “a lot of time was used on copying other teachers’ classroom language and try them in my lesson.”

*We are not going to be speaking English as well as them [class teacher]*, *at least not at the moment*, *but I do think there is more than that [English struggles for teaching]*. *Maybe we can change them [students] if we are good enough [as a teacher]* (*T2*, *interview data*).

Another interesting issue discovered in the process of the interview was that mentor teachers/supervisors played an important role in the CFL teachers’ connection to Australian culture, especially about schooling. As mentioned once in these data, mentors seemed invisible in the CFL teachers’ life, so we focused on them during the interviews. While T3 said her mentor was a “happy person” and “pretty helpful” in giving her some advice, T1 and T2 believed their mentors were “not that helpful.” They viewed the mentors as their “teachers” and were hesitant to talk about their problems. They thought it was from their culture that “mentors are our teacher and should be respected.” “The respect means no questioning even when things are not that great” (T2). “Personality” was mentioned as a key that “makes difference” (T1).

*I know this sounds harsh but to be honest*, *we do not connect*. *She [my mentor]is very busy and I understand that*, *but since she is a mentor*, *doesn’t she think there’s a role to play? I feel that she was waiting for me to ask questions; I was too shy*. *Anyway*, *maybe I’m overthinking* (*T1*, *interview data*).

*Well*, *she doesn’t know much about Chinese*, *and I don’t know what’s going on here*, *so we don’t know what to talk about*. *There are conversations now and then but not that much* (*T2*, *interview data*).

### Environment: Contextual Impact Factors for Chinese as a Foreign Language Teacher’s Identification

While intrapersonal and interpersonal factors were essential for the construct of CFL teacher identity, environmental elements also played in the self-other interaction. Both theses data and interviews revealed the forms of environment and corresponding influences. There was a mention about learning atmosphere as well as resource issues in theses data. “Not satisfying” was the keyword that indicated environmental challenges. Interviewees mentioned “lack of academic” (T1) and “low level of persistence” (T2). The overall learning culture was “unsatisfactory” (T2), and Chinese learning was “the worst” (T3).

*A student was silly*, *and I got angry*. *Instead of helping me to manage him*, *the class teacher said*, *“he’s low and sometimes acts like that to me too*.*” I didn’t argue with her*, *but definitely that’s an excuse*. *Well*, *others were not much better* (*T2*, *interview data*).

*I heard students say*, *“no one likes this subject” or “I’d rather stay home*.*” They don’t see the point of school* (*T1*, *interview data*).

Lack of resources was another issue that reflected contextual challenges. T1 said he could not find anything from the school library for language learning and everything was designed from scratch. T3 mentioned the amount of money she spent on materials and equipment. While T2 supported the idea that “no textbook means flexibility for teachers,” he argued that “there should be a pool available to choose from.”

*I would give CFL teachers something and let them try and see what materials are contributing to what skills of students*. *Then we can build on that and make adjustments*. *Records should be kept of what is useful* (*T2*, *interview data*).

## Discussion

Drawing on theses of 5 years and interviews of three participants of the CFL program, the foregoing part offered a picture of CFL teachers’ construction of teacher identity in Australian contexts. Although the sample size is limited, this study reveals a new angle to approach existing issues. On the whole, our findings support the existing literature (e.g., Chen, 2015; Moloney and Xu, 2015; Han et al., 2019; Han and Han, 2019) that CFL teachers experience various challenges while teaching in foreign contexts, and that their institutional life and teacher identity have to be reorganized according to new social norms. However, instead of examining external factors, such as social environment, pedagogy, and curriculum, we delved into CFL teachers’ internal operation of social interaction and found divergences of self and other in CFL teacher identity development in Australia, which not only includes objective relational process between individuals but also ideological and axiological factors that influence the wholeness of CFL teachers. Such finding has not been explored in depth by previous studies.

In searching for answers to our first research question, CFL teachers’ cultural identity stands out and poses questions of self-other interactions interculturally. First, CFL teachers are equipped with Chinese self that they acquired over the years from social networks in China. The self is a collection of social processes of Chinese characteristics. Viewing Australian students from the Chinese self, CFL teachers may think they are not respected when students do not greet them. There was a mention of “no greeting” (C1) and “ignoring” (C5) in theses data. Such desire for “automatic respect” (C1) comes from the Chinese self where Chinese students’ attitudes to teachers formed the “me” (Mead, 1934; Chen, 2015). Another problem for the CFL teachers is their “authority issue” (T1, C5) that derives from and contributes to their teacher identity (Walkington, 2005; Pinter, 2017). T3 believes authority means “control students’ behavior,”, while T1 thinks “it reflects how much are taught.”. Their English proficiency is a major concern both for class management and learning instruction (Varghese, 2000; Orton, 2016). T1 and T3 struggle to express themselves comprehensively in giving instructions, while T2 focuses on what to say to manage students effectively. This is consistent with the theses data [e.g., “My pronunciation mistake caused a lot of misbehavior” (C3); “I was afraid to give instructions” (C5)]. We then conclude that requirement for Australian students to change their “me” according to CFL teacher’s attitudes of “respect” is not practical, and that expectation of it may lead to dissatisfaction or even frustration (Mead, 1934; Chen, 2015; Orton, 2016).

Most CFL teachers choose to adapt to the Australian culture by changing the self, while T2 wants to change other as well. He believes students’ attitudes toward him as a CFL teacher are manageable and it is part of his “mission.” As a “significant symbol,” language (English) enables adjustments (Mead, 1934). T1 and T3 target class teachers’ fluency and accent, which cause them distress and hopelessness. T1 wonders how he can be as “native” as class teachers with “perfect English,” while T3 thinks she “just cannot do it.” T2 focuses on what he needs for particular purposes and improves his English through “copying what they say and adapting to what I need.” He finds the class teachers’ English was “not hard in vocabulary or grammar,” so he takes notes and prepares his Chinese lessons with them, and “it helped a lot.” This reflects CFL teachers’ growth-mindset for improvement and intercultural sensitivity to discern where and how to improve (Jin, 2017; Dweck and Yeager, 2019). Therefore, it is safe to say that setting an achievable target in “me” is important for the establishment of the teacher identity of CFL teachers.

Adding to previous studies on mentor teachers’ contribution to CFL teachers’ growth (Lai et al., 2015; Wang and Bale, 2019), we find that systematic communication can be lacking because of different backgrounds. T2 reveals that the mentors of the program are Australian teachers who do not know much about Chinese culture, and that he does not know much about Australia. “Having little to talk about” leaves him to struggle to “fit in,” and he admits his personality is “not that open to initiate.” T1 worries about his accent although hopes to talk more with his mentor. Such a gap can be bridged by mentors if they know what their mentees need. T3’s mentor is too busy to be found and talk with, which she thinks reduces connectedness. Interestingly, all the three participants, while possessing questions about their mentor, take them as their “teacher” and show “unconditional respect.” As part of Chinese culture, the self disallows them to respond to mentors with “questioning attitudes” (T2), which leads to a gap of understanding and needs unmet.

The second research question brings us to the functional side of social relationships. In order to have a “complete self,” CFL teachers need “various aspects of the structure of the social process as a whole” (Mead, 1934, p. 144). Lack of interaction with mentors, colleagues, and students leads to insufficient understanding of collective attitudes in contexts. The incomplete self causes confidence issues in teacher identity and limits self-identification (Neilsen and Weinmann, 2020). When the CFL teachers “don’t know what to do with students” (T3) or “wanted to stand up and shout ‘get out of the door”’ (C1), it is the lacking of “reference to the community” (Mead, 1934, p. 142) that disables them. They need a new self that is responsible for social reactions and relationships as “we are one thing to one man and another thing to another” (Mead, 1934, p. 142). Therefore, Australian other provides a new self for CFL teachers to know their audience and how to teach.

Finally, in searching for ways to negotiate between the Chinese self and the Australian other to develop teacher identity, we find that the CFL teachers struggle mostly with acquiring a new self to connect with the new environment. While English proficiency is a major challenge mentioned across the theses and interviews, “purposeful contextual improvement” (T2) seems more achievable and beneficial than trying to be “as local as class teachers” (T1), which may cause distress. Understanding what to use in specific situations allows CFL teachers’ appropriate me to respond to other. Instead of struggling to change accent and fluency, the focus should be on the practical function of English in expressing the Chinese self. Using appropriate English for the communication of Chinese cultural identity is crucial for the construct of teacher identity. Therefore, although English proficiency is key for communication, a specific selection of areas to improve defines the progress of self-identification. CFL teachers’ mindset is also integral for the shaping of their identity (Dweck and Yeager, 2019), as the cases of T2 (growth-mindset of self-development in challenges) and T3 (fixed mindset of validating her low ability and avoiding challenges) attested to.

## Conclusion

This study examines new CFL teacher identity transformation in Australian contexts and factors that lead to various forms of self-other relationships. Coming from Chinese culture and social relationships, the CFL teachers’ Chinese self dominates the way they respond to the new environment. When looking for the Chinese type of respect from Australian students, the Chinese self is not satisfied, and discomfort arises. To connect with the new other, understanding of self is a prerequisite to having proper positioning. Culture shock in this sense is the result of self-other mispositioning. The desire for “unconditional respect” (T3) from the Chinese self is adjusted to “earned respect” (T2), and Australian students’ attitudes toward teachers reshape the “me” of the Chinese self. Furthermore, the relationship between other teachers and students also contributes to the self of CFL teachers, as “me” is a collection of organizational attitudes. Lack of communication with other teachers has not only given the CFL teachers a sense of disconnectedness but has also led to lack of information on how students are viewed and treated in specific contexts.

When approaching Australian other, English was a challenge to the CFL teachers, although they had an English-related degree and sat English tests for the program. Lack of practical use of English leads to confidence issues such as “don’t know what to do” and “afraid of giving instruction” (theses data). Mentor’s help and CFL teacher’s growth-mindset are important in facing this challenge, which may benefit both CFL teacher’s English skills and student’s learning. Another finding is that CFL teachers are shy to seek help even when they know that it is needed. This cultural presentation of Chinese self limits CFL teacher identity development. On this point, we suggest different sessions for programs and the like to explore intercultural communication needs both inside and outside of classrooms and find ways to promote proactiveness of all parties.

Limitations of this study should be highlighted on two fronts: first, the small-scale research focuses on the need of new CFL teachers in Australian schools for identity construction based on a self-other relationship without consideration of personality, which could be a factor influencing self; second, there is a gap between the theses data and interview data, because identity issue is rarely discussed in depth in the former. We suggest that future research should have more CFL teachers included and take their individualities into account for a more comprehensive picture of this issue. Moreover, it is also advisable to look into CFL teachers’ personalities in the future regarding their identity formation.

## Data Availability Statement

The original contributions presented in the study are included in the article/supplementary material, further inquiries can be directed to the corresponding author.

## Ethics Statement

The studies involving human participants were reviewed and approved by Western Sydney University Ethics Committee. The patients/participants provided their written informed consent to participate in this study.

## Author Contributions

Both authors listed have made a substantial, direct, and intellectual contribution to the work, and approved it for publication.

## Conflict of Interest

The authors declare that the research was conducted in the absence of any commercial or financial relationships that could be construed as a potential conflict of interest.

## Publisher’s Note

All claims expressed in this article are solely those of the authors and do not necessarily represent those of their affiliated organizations, or those of the publisher, the editors and the reviewers. Any product that may be evaluated in this article, or claim that may be made by its manufacturer, is not guaranteed or endorsed by the publisher.
